# Clonal dynamics and Stereo-seq resolve origin and phenotypic plasticity of adenosquamous carcinoma

**DOI:** 10.1038/s41698-023-00430-8

**Published:** 2023-08-26

**Authors:** Ruiying Zhao, Yunhua Xu, Yedan Chen, Jiajun Zhang, Fei Teng, Sha Liao, Shengnan Chen, Qian Wu, Chan Xiang, Jiaohui Pang, Zhanxian Shang, Jikai Zhao, Hairong Bao, Hua Bao, Yang Shao, Shun Lu, Yuchen Han

**Affiliations:** 1 Department of Pathology, Shanghai Chest Hospital, Shanghai Jiao Tong University School of Medicine, Shanghai, 200030 PR China; 2 Shanghai Lung Cancer Center, Shanghai Chest Hospital, Shanghai Jiao Tong University School of Medicine, Shanghai, 200030 PR China; 3 Geneseeq Research Institute, Nanjing Geneseeq Technology Inc., Nanjing, 210032 PR China; 4 BGI Research, Chongqing, 401329 PR China; 5 BGI Research, Shenzhen, 518083 PR China; 6 School of Public Health, Nanjing Medical University, Nanjing, 211166 PR China

**Keywords:** Non-small-cell lung cancer, Cancer genomics

## Abstract

The genomic origin and development of the biphasic lung adenosquamous carcinoma (ASC) remain inconclusive. Here, we derived potential evolutionary trajectory of ASC through whole-exome sequencing, Stereo-seq, and patient-derived xenografts. We showed that *EGFR* and *MET* activating mutations were the main drivers in ASCs. Phylogenetically, these drivers and passenger mutations found in both components were trunk clonal events, confirming monoclonal origination. Comparison of multiple lesions also revealed closer genomic distance between lymph node metastases and the ASC component with the same phenotype. However, as mutational signatures of *EGFR*-positive lung squamous carcinomas (LUSCs) were more comparable to *EGFR*-positive ASCs than to wild-type LUSCs, we postulated different origination of these LUSCs, with ASC being the potential intermediate state of driver-positive LUSCs. Spatial transcriptomic profiling inferred transformation from adenocarcinoma to squamous cell carcinoma, which was then histologically captured in vivo. Together, our results explained the development of ASC and provided insights into future clinical decisions.

## Introduction

Adenosquamous carcinoma (ASC) is a rare entity of non-small-cell lung cancer (NSCLC) with an incidence of 0.4% to 4% of all lung cancers^1^. It is characterized by a biphasic morphology containing at least 10% adenocarcinoma (AC) and squamous cell carcinoma (SCC) components^2^. Due to the rarity of the disease, evidence to guide clinical decisions of ASC is lacking. Earlier studies showed that resected ASC was more aggressive and had a higher possibility of metastatic seeding than pure AC or SCC, resulting in a poor prognosis^3–6^. Surgical resection is the mainstay treatment for early-stage ASCs, but no consensus exists on the therapy regimen in adjuvant or advanced settings^7^. In the past decade, growing molecular investigations have discovered alterations in driver oncogenes in ASC, such as *EGFR* and *KRAS*, indicating that recurrent or advanced ASCs could also benefit from targeted therapies. Several studies revealed that treating *EGFR*-positive ASC with first- or third-generation TKIs produced similar efficacy as typical lung adenocarcinomas (LUAD)^8–10^.

Presenting mixed glandular and squamous phenotypes, how ASCs come about remained mysterious. Early animal studies established that the two components originate from different progenitor cells that ultimately migrate and infiltrate into each other^11^. However, later immunohistochemical (IHC) evidence found similar expression of squamous markers in both components and argued that their relationship might be more intertwined than simply being a spatial mixture of two distinct subtypes^12,13^. Recent genomic studies of micro- or macrodissected ASCs further revealed shared mutations between AC and SCC, including *EGFR* and *KRAS*, suggesting ASC are potentially monoclonal origin^1,14,15^. These studies primarily focused on hotspot mutations or used hotspot sequencing panels. Information from broader genomic areas is crucial in understanding clonal relationships and evolutionary trajectories.

In this study, we microdissected surgical ASC samples and performed whole-exome sequencing to identify genomic aberrations and mutational signatures of primary ASCs. By reconstructing clonal phylogenies using primary and lymph node metastases, we depicted the clonal relationships of AC and SCC components to support the monoclonal origin theory of ASC. We also demonstrated histologic transformation of ASC in a preliminary xenograft model, providing insights into the cells of origin and potential lineage plasticity of the disease.

## Results

### Description of patient cohorts and study design

Total of 33 ASC patients, including 12 female and 21 male patients, underwent surgical resection at our center, and their archived FFPE samples were collected for this study (Supplementary Fig. 1). Fourteen patients (42%) had lymph node metastases, and one patient had pleural metastases. In addition, three patients had separate non-ASC primary cancers. Among these, two were known smokers, two were former smokers, and two were unknown. Other 27 patients (82%) reported no history of cigarette smoking (Table 1). Overall, primary ASCs contained 15–80% of squamous components according to their histology. Roughly two-thirds of these (21/33) displayed balanced AC and SCC components (comprising 40–60% squamous proportion). To investigate the molecular mechanism of ASC, these primary resected tumors were microdissected to separate AC and SCC components, including two patients whose AC components were further dissected into high/moderate-differentiation and low-differentiation areas.

**Table 1:** Clinical characteristics of microdissected adenosquamous carcinoma and *EGFR*-positive lung squamous cell carcinoma patients in this study.

	Adenosquamous carcinoma	EGFR-positive lung squamous cell carcinoma
*n*	33	7
Age		
Median	65	62
Range	44–78	37–72
Sex		
Female	12 (36%)	2 (29%)
Male	21 (63%)	5 (71%)
Smoking history		
Yes	2 (6%)	1 (14%)
Former	2 (6%)	0
No	27 (82%)	6 (86%)
NA	2 (6%)	0
Disease stage at diagnosis		
IA2	3 (9%)	0
IA3	2 (6%)	0
IB	6 (18%)	1 (14%)
IIA	2 (6%)	0
IIB	10 (30%)	3 (43%)
IIIA	7 (21%)	1 (14%)
IIIB	1 (3%)	2 (29%)
IIIC	1 (3%)	0
IV	1 (3%)	0
Lymph node metastasis		
No	19 (58%)	4 (57%)
Yes	14 (42%)	3 (43%)

Furthermore, we compared 33 Asian LUADs 25 pure LUADs from the TCGA database and our internal database. Of these, 14 were *EGFR*-positive. To reconstruct possible relations between the *EGFR*-positive tumors, we also sequenced seven *EGFR*-positive resected LUSCs and included 15 *EGFR*-wild-type LUSCs from existing databases (Supplementary Fig. 1).

To evaluate the efficacy of EGFR-TKIs in *EGFR*-positive lung cancer patients, we included 160 LUAD, 52 ASC, and 65 LUSC patients who were treated with any EGFR-TKI monotherapy or combination therapies in the clinic (Supplementary Table 2). Overall, 131/160 (82%) LUAD patients received EGFR-TKI as first-line treatment, 118 (74%) of which were treated with first-generation TKIs. In *EGFR*-positive ASCs and LUSCs, TKIs were administered slightly later during therapy, with 25/52 (48%) and 44/65 (68%) patients receiving TKIs as first-line treatment, respectively. In addition, 18/52 (35%) ASC and 19/65 (29%) LUSC patients received TKIs as second-line treatment.

### Genomic landscape of microdissected primary ASCs

**Fig. 1:**
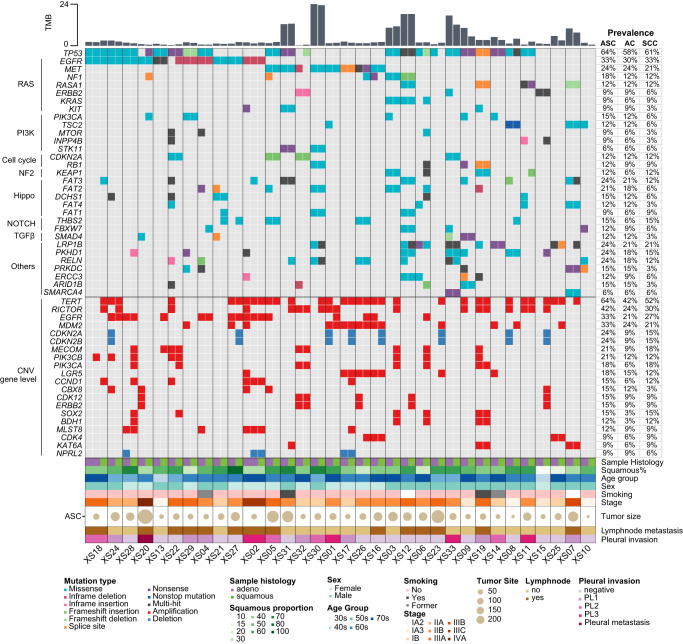
Genomic alterations in microdissected AC and SCC components of ASC. Mutational landscape of key oncogenic genes and gene-level copy number variations are shown. Prevalence of alterations was shown in AC and SCC components, and in ASC.

The prevalence of genetic alterations was calculated for ASC as a single disease (detected in either AC or SCC), and for each component separately. Frequently mutated genes were shown in their corresponding oncogenic pathways. We first assessed known NSCLC driver events in the ASCs. Activating *EGFR* mutations were found in 11/33 patients (33%), including five with exon-19 deletion, five with L858R, and one with L861Q. All patients, except one patient, carried *EGFR* mutations in both AC and SCC components*. MET* exon 14 skipping was identified in both components of five patients (5/33, 15%) (XS01, XS05, XS17, XS26, XS30), which was more frequently observed than in pure LUAD^16^. In addition, three patients (9%) harbored *KRAS* G12C/D mutations (one G12D found only in SCC), and one had *ERBB2* exon 20 insertion in both AC and SCC (XS32). Importantly, these driver events were mutually exclusive from each other (Fig. 1). Other Ras-pathway genes, such as *NF1* (6/33) and *KIT* (3/33), were also detected. In the PI3K pathway, mutations in *PIK3CA* and *TSC2* were observed in four AC component (4/33, 12%) and slightly lower in SCC (6%). Altered cell cycle pathway genes, *CDKN2A* and *RB1*, were found at balanced prevalence in both components (both 4/33, 12%). Moreover, mutations in *FAT3*, *LPP1B*, *PKHD1*, *RELN* (8/33, 24%, respectively), and *FAT2* (7/33, 21%), and gain of *TERT* (21/33, 64%), *RICTOR* (14/33, 42%), *EGFR* (11/33, 33%), and *MDM2* (11/33, 33%) were also frequently detected in ASC (Fig. 1).

**Fig. 2:**
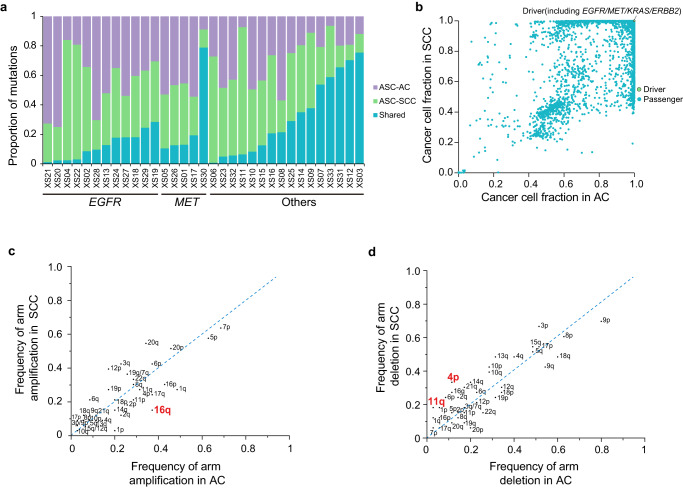
Comparison of AC and SCC components in ASC. **a** Proportion of shared and unique mutations in AC and SCC components of each ASC patient. **b** Comparison of cancer cell fractions of shared mutations, where the green dots represent driver mutations and blue dots represent passenger mutations. **c**, **d** Arm-level amplification and arm-level deletion events.

Overall, genomic profiles were similar between AC and SCC components (no statistical significance by fisher’s exact test). Nevertheless, most tumors present shared driver and passenger mutations. For *EGFR*-driven ASCs, an average of 12.2% somatic mutations were shared between AC and SCC (1.1–28.4%). For *MET*-driving ASCs, an average of 26.8% of mutations were shared (10.5–78.9%). Other tumors also share various proportions of mutations ranging from 0.7% to 75.4%, indicating possible same genomic origins of AC and SCC components (Fig. 2a and Supplementary Fig. 2). No difference was observed in the distribution of ccf for all shared mutations between AC and SCC components. Importantly, most driver mutations have estimated ccf values greater than 0.99 in both components, indicating they were potentially major clones that drove the development and progression of the tumor (Fig. 2b). For arm-level copy number alterations, SCC components demonstrated significant (*P* = 0.041) and marginal significant (*P* = 0.051) enrichment of chromosome 4p loss and 11q loss, respectively, whereas AC components showed enrichment of 16q amplification (Fig. 2c, d). Further, we assessed potential amplification of the *NKX2-1* gene, which encodes for TTF1, a biomarker that distinguishes adenocarcinomas. We found that 15 out of the 33 ASC patients (45%) had shallow amplification in segments covering the *NKX2-1* gene region, but not *NKX2-1*-specific deep amplification. Of these 15 patients, 10 had *NKX2-1* amplification detected only in the AC compartment, 2 only in the SCC compartment, and 3 in both compartments (Supplementary Table 1).

### Genomic features of primary and metastatic ASCs

**Fig. 3:**
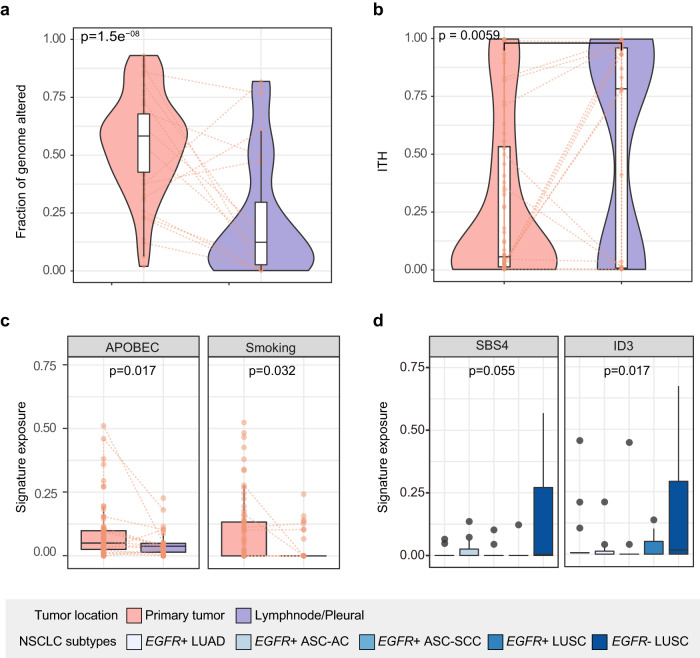
Genomic features of primary and metastatic ASCs. **a** Fraction of genome altered compared between primary (pink) and lymph node or pleural metastases (purple) indicating genomic instability levels. **b** Intratumoral heterogeneity score compared between primary (pink) and lymph node or pleural metastases (purple) indicating the proportion of subclonal mutations. **c** APOBEC and smoking-associated signatures compared between primary (pink) and lymph node or pleural metastases (purple). **d** Smoking-associated signatures compared between *EGFR*-positive NSCLC subtypes (LUADs, ASCs, and LUSCs) and *EGFR*-negative LUSCs. Two-sided *P* values were compared using Wilcoxon rank-sum test. The center lines of the boxes represent the median value of each covariate. The lower and upper edges of the boxes correspond to the first and third quartiles, and whiskers defines the range within 1.5 times the interquartile range (IQR) from the edges of the boxplot. Dots indicate outliers, defined as data points that fall outside 1.5 times the IQR.

Next, we compared genomic instability (measured by fraction of genome altered, FGA) and proportion of clonal and subclonal mutations (measured by intratumoral heterogeneity (ITH) score) in primary and metastatic tumors. We observed significantly higher proportion of FGA in primary ASCs than lymph node or pleural metastases (*P* < 0.001 by Wilcoxon rank-sum test) (Fig. 3a). Instability was predicted by higher proportion of APOBEC-associated mutations in the primary tumor (*P* = 0.017) (Fig. 3c). Despite similar tumor mutational burden, metastatic sites displayed significantly higher ITH than primary ASCs (*P* = 0.0059 by Wilcoxon rank-sum test) and the similar result was observed in the *EGFR-*positive ASCs subgroup (*P* = 0.018) (Fig. 3b and Supplementary Fig. 3a, b). Tobacco smoking contributed to significantly higher proportion of mutations in *EGFR*-wild-type LUSCs than in *EGFR*-positive subtypes (SBS4 and ID3) (SBS4, *P* = 0.055; ID3, *P* = 0.017) (Fig. 3d). Overall, *EGFR*-positive LUSCs presented similar mutation signature profiles and ITH to *EGFR*-positive ASCs (Supplementary Figs. 3c, d and 4a). In *MET* exon 14 skipping tumors, however, SCC was dominated by signatures associated with defective DNA mismatch repair, contributing to roughly 25% of SCC mutations, which was higher than corresponding AC component and significantly higher than that in *MET*-positive LUADs. Conversely, APOBEC and smoking-related mutations were more enriched in LUADs but did not mainly induce mutations in ASCs (Supplementary Fig. 4b).

### Reconstruction of tumor evolution in ASCs

**Fig. 4:**
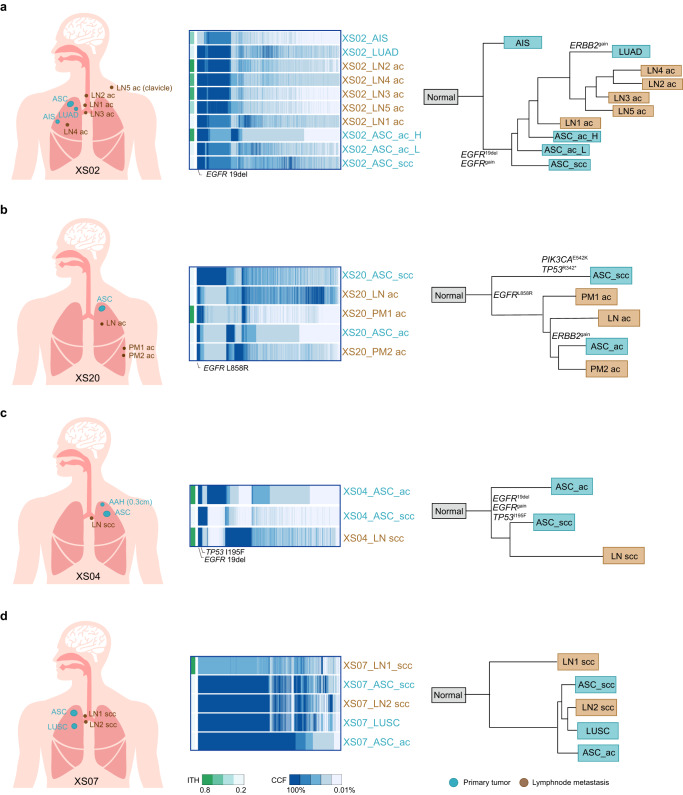
Evolutionary phylogeny depicting relationships of phenotypic different lymph node metastases and primary ASC components. Left panel is schematic diagrams showing the positions of lesions taken from **a** Patient XS02, **b** Patient XS20, **c** Patient XS04, and **d** Patient XS07 with labeled histological subtypes. The middle panel is landscapes of shared and unique genetic mutations and corresponding cancer cell fractions, and the right panel shows phylogenic trees reconstructed with maximum parsimony with key lung cancer alterations labeled. Histological subtypes: LUAD lung adenocarcinoma, ASC adenosquamous carcinoma, AIS adenocarcinoma in situ, LN lymph node metastases, PM pleural metastases, AF allele frequency, ITH Intratumoral heterogeneity.

The above genomic features supported the monoclonal origination of ASC with different features in metastatic sites. To further explore the evolution routes of ASC, we reconstructed phylogeny trees based on single-nucleotide variants and CNVs from primary and lymph node samples with the maximum parsimony approach. Among the study patients, 13 had lymph node (LN) or pleural metastases (PM), and phylogenetic distance of these metastases were found to be closer to the ASC component with same histology. For example, Patient XS02 had primary ASC, primary LUAD and adenocarcinoma in situ (AIS) at diagnosis. The ASC lesion contained 30% SCC proportion, whereas the AC component was further separated into highly/moderate- and poorly-differentiated parts. *EGFR* exon-19 deletion was considered clonal event at the trunk of tumor development. The three microdissected ASC samples were clustered closely in the phylogenetic route, suggesting shared evolutionary root. Meanwhile, the *EGFR*-positive primary ADC also carried gain of *ERBB2* and a separate phylogenic clade, indicating potential early seeding of clonal *EGFR* (Fig. 4a). Similarly, *EGFR* L858R was one of the clonal mutations and found in all samples of Patient XS20, whereas subclonal *PIK3CA* E542K and *TP53* R342 truncating mutations were only detected in the SCC component. Resulted phylogeny tree also indicated separate routes of histologically different tumors (Fig. 4b). In addition to supporting the monoclonal origination of AC and SCC components, these evolutionary architectures also implied that the two components might evolve separately. These patients’ LN metastases showed AC phenotype and were on the same branch as AC component of ASC (Fig. 4a, b and Supplementary Fig. 5). Conversely, if the LN metastases presented SCC phenotype, they were evolutionarily closer to the SCC component of primary ASC (Fig. 4c, d and Supplementary Fig. 5), suggesting that they might be seeded by these tumor cells.

### Deciphering dynamic transition between histological components in ASCs

**Fig. 5:**
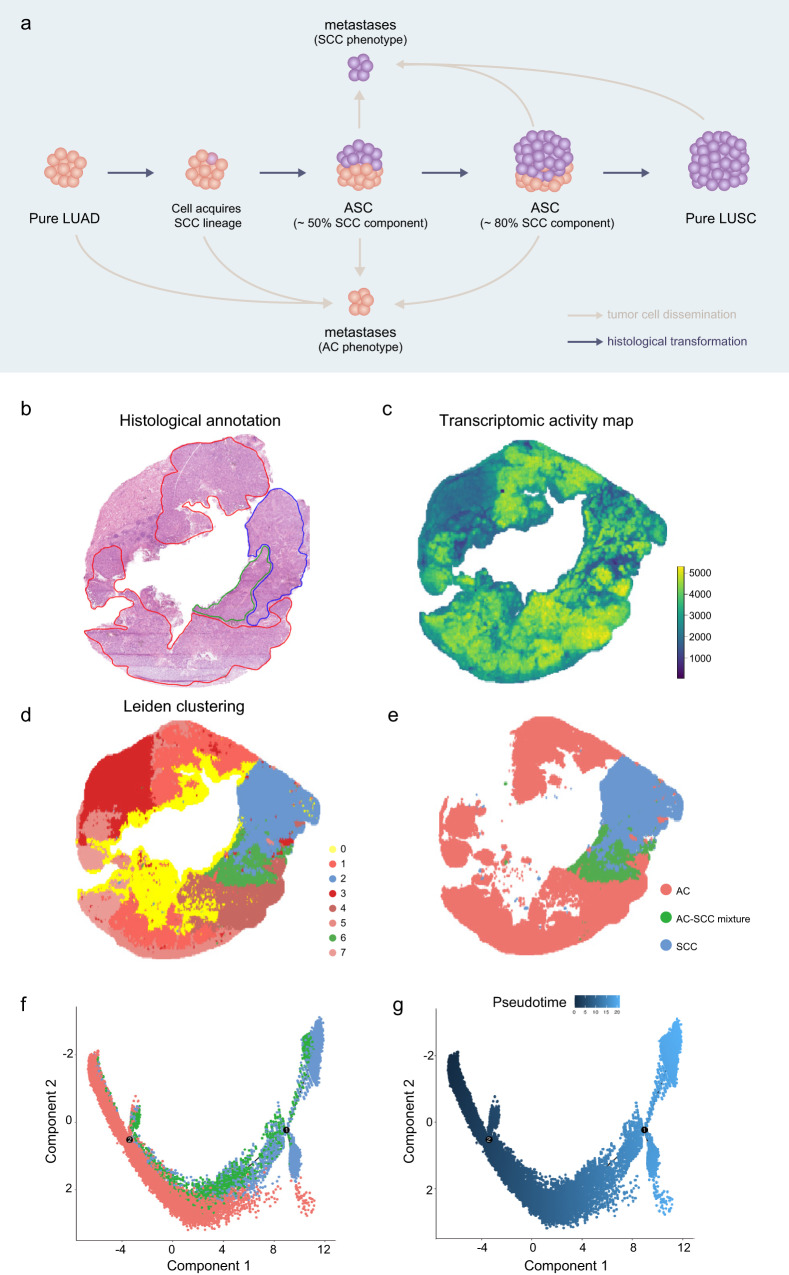
Spatial transcriptomic profile illustrating biphasic phenotype of ASC. **a** Illustration of the proposed concept of ASC’s development, dissemination, and histological transformation. **b** Histological annotation of treatment-naïve ASC tumor. Area circled by red marker showed AC phenotype, blue displayed SCC phenotype. Green enclosed area presented mixed AC and SCC phenotypes. **c** Spatial transcriptomic activity map of an immediate adjacent section to the H&E-stained section. Colors indicate the number of genes expressed. **d** Unsupervised clustering of spatially resolved profiles of genes with Leiden clustering into eight clusters indicated by different colors. **e** Clusters 1, 4, 5, and 7 were grouped as AC. Cluster 2 was identified as SCC. Cluster 6 was identified as AC-SCC mixture. **f** Selected clusters from (**e**) were analyzed by Monocle2. Red dots indicated AC, blue dots indicated SCC, and green dots indicated dual phenotypes. **g** Pseudotime analysis showed the transition from AC (dark blue) towards SCC (light blue).

In the clinic, histological transformation is often observed among lung cancer subtypes. Based on our genomic and phylogenetic evidence, we speculated that ASC was an intermediate state during potential transformation from AC to SCC. Different SCC proportion of ASC reported with the clinical diagnosis of ASC then reflects the extent of squamous cell transition. During the tumor growth and development, AC or SCC cells could each disseminate and give rise to metastases of either phenotype (Fig. 5a).

To validate the evolutional concept between histomorphological components of AC and SCC, we applied Stereo-seq to obtain spatially resolved RNA profiles in 10μm tissue section from one prospectively collected resected ASC. Histological types were confirmed by pathologists by H&E and IHC staining to ensure that components of AC and SCC were included (Fig. 5b). We observed distinct areas presenting either AC or SCC phenotype, as well as an area of mixed phenotypes. An immediate adjacent cryo-section was prepared for detection of spatially resolved RNA profile with Stereo-seq as previously mentioned^17^. Analysis was performed with binned (bin 100,100 × 100 DNB bins, 50-μm diameter) Stereo-seq data. The distribution of gene counts shown in Fig. 5c revealed higher transcription activity in AC areas than in SCC or AC-SCC mixture areas. Unsupervised clustering on binned data showed eight different cell clusters (Fig. 5d). Referring to the histological annotations by H&E staining, Clusters 1, 4, 5, and 7 were annotated as AC, Cluster 2 as SCC, and Cluster 6 presenting dual phenotypes of AC and SCC. Using these phenotypic annotated clusters, we then constructed a transcriptional trajectory using Monocle2 to elucidate the evolution path (Fig. 5f). The resulting trajectory showed AC and SCC formed two different branches, with the AC-SCC dual phenotype cluster (Cluster 6) located in between. Importantly, pseudotime analysis elucidated the transition route from AC through dual phenotypes to SCC, supporting the proposed concept of ASC’s evolution direction (Fig. 5g).

### Squamous transition observed in ASC-derived xenograft model

**Fig. 6:**
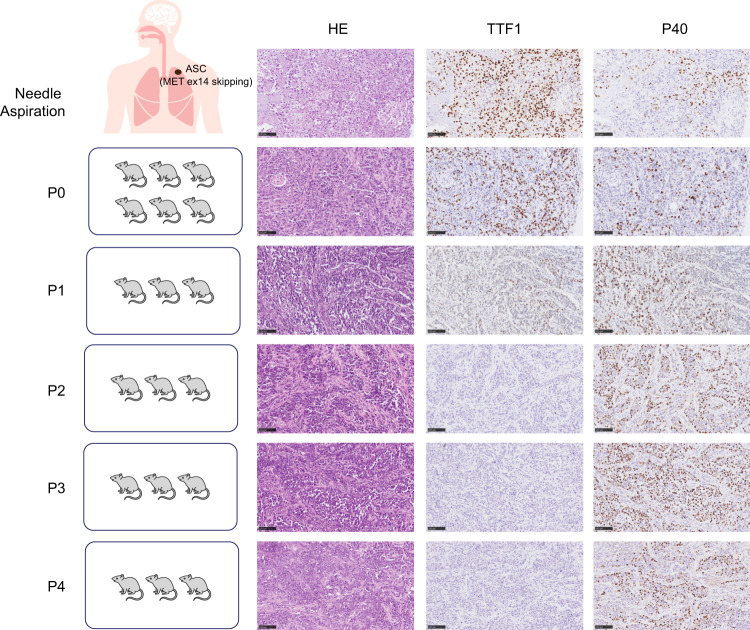
Xenograft model of ASC. Tumor tissue sample of a patient diagnosed with ASC of *MET* exon 14 skipping mutation was taken and subcutaneously grafted in female NOD SCID mice. A total of four passages were generated by serial implantation. Tumor morphology was immunohistochemically evaluated for the expression of TTF1 and P40 markers.

To further explore the phylogeny of ASC, patient-derived ASC cells were inoculated in female mice. The patient was diagnosed with advanced ASC with *MET* ex14 skipping mutation. The histology of tumors was compared before and after tumor inoculation. Both patient tissue and Passage 0 (P0) tumors showed strong diffuse expression of TTF1 and partial expression of P40 in different cell populations. Notably, starting from the P1 generation, TTF1 expression decreased markedly while P40 expression became more dominant. In P2, P3 and P4 tumors, there were hardly any TTF1 expressed. In contrast, more than 90% of the tumor regions were positive for P40, implying near-completion of squamous transition (Fig. 6). As TTF1 is a primary maker for LUADs, expressed in up to 80% cases^18^, and P40 is consistently expressed in squamous cells^19^, the phenotype of P0 indicated a mixture of glandular and squamous cells, whereas P1 captured the transition into an SCC predominant phenotype due to decreased AC component. Generations after P2 lost adenocarcinoma features and became pure squamous cells. Interestingly, no treatment was administered during the grafting and passaging processes, indicating that predetermined cell fates or natural selection forces driving histological transformation. To validate evolutionary relationships between patient tumor and the histologically transformed mouse xenografts, we reconstructed phylogenetic trees of previously established PDX from a *MET* ex14 skipping ASC patient^20^. Specifically, untreated patient tumor (TTF1 + /P40 + ) and P3 of the untreated PDX tumor (TTF1−/P40+) was used for analysis. We found that 71.0% of the mutations in P3 were also present in the patient’s primary tumor, including *MET* exon 14 skipping, *TP53* H193R, and *BRCA1* Q1240* nonsense mutations (Supplementary Fig. 6a). Importantly, these shared driver and passenger mutations were identified as trunk clonal events with high ccf values in both samples, whereas branch mutations had much lower ccf values (Supplementary Fig. 6b). These indicated that although tumor cells had acquired squamous phenotypes in the P3 mice, their genetic profiles still represented that from their clonal origins.

### Efficacy of EGFR-TKIs in *EGFR*-positive LUADs, ASCs, and LUSCs

**Fig. 7:**
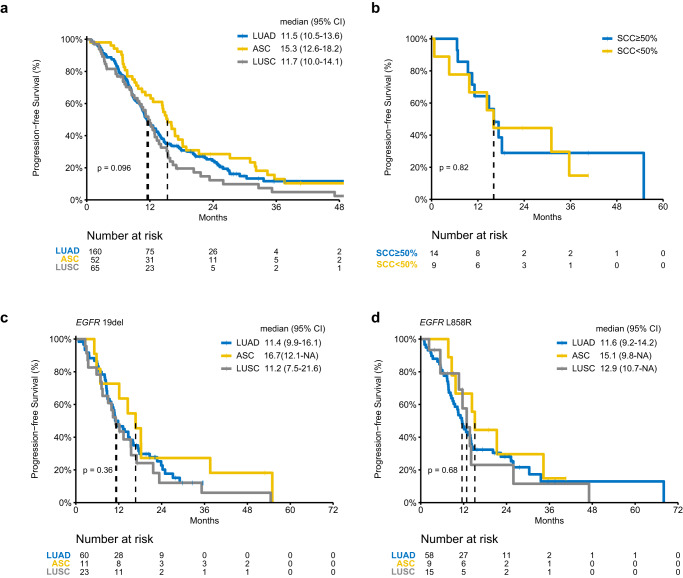
Efficacy of EGFR-TKIs in LUAD, ASC, and LUSC. Kaplan–Meier curves estimate the progression-free survival of **a** LUAD, ASC, and LUSC treated with EGFR-TKIs, **b** resected ASC with evaluable SCC proportion above and below 50% upon EGFR-TKI treatment, **c** LUAD, ASC, and LUSC patients with *EGFR* exon-19 deletion receiving first-generation EGFR-TKIs as first-line treatment, and **d** LUAD, ASC, and LUSC patients with *EGFR* L858R on receiving first-generation EGFR-TKIs as first-line treatment. Two-sided *P* values were calculated using the log-rank test.

As approximately 30% of ASCs are *EGFR*-positive, we wondered how they respond to EGFR-TKIs especially in comparison to *EGFR*-positive LUADs and EGFR-positive LUSCs. A separate *EGFR*-positive cohort of 160 confirmed pure LUAD, 52 ASC, and 65 confirmed pure LUSC patients were retrospectively collected (Supplementary Table 2). Overall, the efficacy of EGFR-TKIs was similar among the three subtypes. Median PFS (mPFS) of ASC (15.3 months, 95% CI 12.6–18.2) was slightly better than other subtypes without statistical significance (LUAD, 11.5 months, 95% CI 10.5–13.6; LUSC 12.0 months, 95% CI 10.0–15.2) (Fig. 7a). Of 23 resected ASCs from this cohort, tumors were considered SCC predominant if AC component was less than 50%. The proportion of SCC components did not significantly affect TKI efficacy (Fig. 7b). For patients receiving first-line treatment of first-generation TKIs, similar outcomes were observed among three subtypes in both *EGFR* L858R and *EGFR* exon-19 deletion subgroups (Fig. 7c, d).

## Discussion

Understanding the molecular mechanisms and evolutionary trajectory of ASC is crucial in facilitating clinical decision of the disease. In this study, we found that microdissected ASCs present shared oncogenic drivers and other genomic alterations.

In our cohort, 33% ASC patients are positive for *EGFR* sensitizing mutations in both AC and SCC components. Depending on the ethnicity and cohort sizes, reported prevalence of *EGFR* mutation in ASC varied from 10 to up to 80%^10,14,15,21^. TKI efficacy results also reinforced the importance of *EGFR* mutation testing in ASC and LUSC. We found that the efficacy of first-line TKI was similar among three *EGFR*-positive NSCLC subtypes, suggesting that TKI should still be considered as a first-line regimen in all naïve *EGFR-*positive NSCLC patients. Moreover, the occurrence of *MET* exon 14 skipping mutations in our cohort was much higher than typical LUADs, suggesting possible clinical benefit from MET inhibitors, such as crizotinib, tepotinib and capmatinib. To our knowledge, no *MET*-positive ASC has been reported yet, probably because of the dual effect of *MET* being an infrequent lung cancer driver and ASC being a rare lung cancer subtype. The choice of testing panels might also affect the chance of detecting *MET* exon 14 skipping mutations.

From a genomic standpoint, shared mutations can be helpful in delineating the clonal relations of cancer^22^. Earlier studies found identical oncogenic drivers, such as *EGFR* and *KRAS*, in both components of ASC and concluded that these tumors are of monoclonal origin^10,15^. However, *EGFR* is a featured NSCLC driver with a high mutation rate and sensitive mutations of *EGFR* are almost always clonal events that involve in tumor initiation and development^23^. Therefore, it might not be conclusive enough to claim monoclonality simply based on shared oncogenic drivers. Information on passenger mutations is also crucial to extrapolate clonal relationships. Recent studies took advantage of next-generation sequencing and identified shared driver and passenger mutations in both AC and SCC components that supported the monoclonal theory more forcefully^1,14^. Here, we provide additional evidence from reconstructed tumor phylogenies that ASC components are closely related along the evolution trajectories. Besides, our mutational signature analyses suggested that smoking is less associated with ASCs and only four patients were ever smokers in our ASC cohort, similar to a previous report^14^. Importantly, the smoking-associated mutation signature (SBS4) was neither the primary contributor in *EGFR-*positive LUAD nor *EGFR-*positive LUSC, especially in comparison to *EGFR* wild-type LUSCs, indicating that *EGFR-*positive LUSCs might be of different origins as other LUSCs. Their responsiveness to EGFR-TKIs also implied that they might be fundamentally different from other LUSCs.

Despite the above genomic findings, how ASCs establish the biphasic phenotype and why tumors reveal different squamous cell proportions remained mysterious. For example, one transcriptomic study suggested that ASC was a complicated mix of AC and SCC tumors of discriminant expression features^24^. Controversy of ASCs’ cell of origin raised from the “collision theory”, which argued that AC and SCC were from different progenitors that developed separately and then co-invaded and mingled spatially^11^. This is supported by the distinct cells of origin hypothesized for NSCLC subtypes: pure LUSCs usually arise proximally from squamous epithelial tissues and tracheobronchial basal progenitors, whereas LUADs are peripherally originated from the small bronchi, bronchioles or alveoli^25^. However, SCC might also arise from glandular cells to protect the respiratory epithelium from exposure to environmental stress like tobacco and chronic inflammation^26^. In fact, metaplastic changes and phenotypic interconversion are prevalent among lung cancer subtypes. Notably, most of such transformation were induced by drug treatment that led to acquired resistance, including squamous transformation of *EGFR*-positive LUADs^27–35^, transformation of ASCs into LUSCs, small cell lung cancers, or sarcomas (through epithelial–mesenchymal transition) and from LUADs into ASCs^27,36–38^. Interestingly, in one recurrent ASC, both pure LUSC and pure LUAD metastases were discovered, implying potential dissemination from a common ASC origin^39^.

Being one of the recently proposed new hallmarks of cancer, phenotypic plasticity allows tumor cells to escape anti-proliferative end states of differentiation and aids tumor invasion and metastasis^40,41^. Growing clinical evidence have corroborated lineage transition as a potential mechanism of ASC, where certain adenocarcinoma cells might acquire squamous cell fate. However, as phenotypic transformation can be transient, bidirectional, and affected by multiple endogenous and exogenous factors, its onset is hard to capture and delicate lineage tracking strategies are required^41^. One such functional genetics study found that in *Kras/Lkb1* mice, *Lkb1*-defiency in LUAD reduces collagen disposition and triggers extracellular matrix remodeling, which in turn upregulates the squamous cell lineage survival gene p63 to facilitate phenotypic trans-differentiation^42^. Importantly, if genetic labeling experiments are not possible due to scarce samples, somatic mutations can also be used as lineage markers to provide higher scalability and faster predictions of lineage tracing. This method relies on computational algorithms to reconstruct evolution trees and is useful in studying the tumor cells^43,44^. Therefore, we used a combination of computational approaches and PDX model to provide supportive evidence of fate transitions. Upon analyzing the genetic profiles, we found that the SCC cells have a common clonal origin with AC cells in both human cohort and PDX model. By spatially resolved the expression profiles of ASC, the projection of the different expression clusters overlapped correspondingly to distinct histological areas of the tumor, where the biphasic area was captured as a separate cluster. Intriguingly, pseudotime trajectory inferred a transition from the adenocarcinoma phenotype to squamous phenotype. This direction of phenotypic change was further confirmed in our preliminary PDX model. A gradual squamous transition of ASC was observed over four passages of xenograft tumors. Our results suggested that ASC might be an intermediate phase after certain adenocarcinoma cells acquire squamous cell fate. As squamous cells are found to survive better than adenocarcinoma cells^45^, they might gain the aggressive features and out-compete the remaining adenocarcinoma cells to complete the phenotypic transition. The squamous proportion reported with clinical diagnosis then likely reflects the degree of squamous transition. Our proposed concept then effectively connected the seemingly opposite theories of ASC’s origination. However, this hypothesis needs further in vitro studies with genetic labels to establish more conclusive findings. Importantly, the presented transition was not therapy-driven, implying potential endogenous selection stress, such as certain genetic predisposition, accumulated reactive oxygen species or inflammation, that might activate and accelerate the trans-differentiation of tumor cells^40,46^. Nevertheless, it would be informative to evaluate treatment-induced pressure to better understand the mechanisms of phenotypic changes.

There are some limitations of this study. First, microdissection was performed based on morphology and visible histologic biomarkers. As NGS is sensitive to detecting molecular changes, mutation-based clonal origin analyses could be confounded by co-infiltrating ASC components inseparable at the pathology level. Additional clues are required for depicting the clonal relationships of ASC. Moreover, the diagnosis of ASC requires simultaneous detection of both histologic subtypes from large tissue specimens. Due to the inextricable sampling bias from small biopsies, preoperative or advanced ASCs might be misdiagnosed as pure LUAD or LUSC. Therefore, studies also recommended assessment of additional biopsies to distinguish ASC from LUSC, especially for *EGFR*-positive tumors^47^. Lastly, due to the paucity of *MET-*positive ASCs, we only depicted clonal relationships of *EGFR*-positive tumors by phylogeny reconstruction. Alternatively, for *MET-*positive ASCs, we presented the squamous transition process through in vivo mouse model to elucidate the potential developmental mechanism of ASC. In the future, establishing *EGFR*-positive xenograft models can be instructive for mechanisms of phenotypic changes in *EGFR*-ASCs, for example, whether they follow different transition dynamics or rely on stronger selection forces, like TKI treatments. Epigenetic modifications and expression profiles of ASC should also be explored to better understand the presented lineage infidelity. Therefore, well-designed functional experiments are essential to trace and identify cell progeny in ASCs.

In conclusion, we have found that AC and SCC components of ASC share similar mutational and copy number alteration profiles. By phylogenetic analyses, we confirmed that these components shared clonal origins and could disseminate separately during tumor evolution. We also demonstrated the histological transformation of ASC into squamous phenotype, suggesting that driver*-*positive LUSCs could originate from AC cells. In the clinic, *EGFR-*positive LUSC patients have benefited from first-line TKI treatment, highlighting the importance of molecular testing in all lung cancer subtypes to facilitate decision-making.

## Methods

### Patients and sample details

Thirty-three patients diagnosed with primary lung adenosquamous carcinoma (ASC) underwent surgical resection at Shanghai Chest Hospital between September 2017 and November 2019. Of these 33 patients, 14 had lymph node metastasis and three had multiple primary lesions of pure adenocarcinoma or SCC at diagnosis, which were surgically resected. Primary ASC samples were marked for adenocarcinoma and squamous components based on Hematoxylin and Eosin (H&E) staining and expression of IHC markers, including TTF1, Napsin A, p40, and CK5/6 and rigorously confirmed by two experienced pathologists. Then, laser-capture microdissection (LCM) was performed on all ASC sample sections using the ArcturusXT™ LCM system (Applied Biosystems). In addition, surgical samples from seven patients diagnosed with *EGFR*-positive primary LUSC were collected for sequencing to compare their genomic features with other *EGFR*-positive subtypes and *EGFR* wild-type LUSCs. This study has been reviewed and approved by the Shanghai Chest Hospital Research Ethics Committee (KS1851). All patients have signed written informed consent forms for genomic profiling.

### Whole-exome library preparation and sequencing

Whole-exome sequencing was performed on microdissected tumors and EGFR-positive primary LUSCs. Genomic DNA from FFPE samples or the whole blood control sample was extracted using QIAamp DNA FFPE Tissue Kit (Qiagen) and DNeasy Blood and tissue kit (Qiagen), respectively, and fragmented by M220 Focused-ultrasonicator (Covaris) into ~250 bp. A whole-genome library was prepared using KAPA Hyper Prep Kit (KAPA Biosystems). Whole-exome capture was performed using the xGen™ Exome Hybridization Panel (Integrated DNA Technologies, Inc.) according to the manufacturer’s protocol. Captured libraries were amplified with Illumina p5 (5’-AAT GAT ACG GCG ACC ACC GA-3’) and p7 primers (5’-CAA GCA GAA GAC GGC ATA CGA GAT-3’) in KAPA HiFi HotStart ReadyMix (KAPA Biosystems) and purified using Agencourt AMPure XP beads. Enriched libraries were sequenced using the Illumina HiSeq 4000 platform as paired 125 bp reads according to the manufacturer’s instructions. The mean coverage depth of WES was 97× and 56× for tumor and normal samples, respectively.

### Variant filtering and mutation calling

Analysis of sequencing data was performed using an automated pipeline as previously described^48^. Briefly, Trimmomatic was used to trim adapters and remove low quality reads (quality reading below 20) or N bases from FASTQ files. Burrows-Wheeler Aligner (BWA) was then used to align clean paired-end reads to the reference human genome (hs37d5). PCR deduplication was performed using Picard and indel realignment and base quality score recalibration were performed using Genome Analysis Toolkit (GATK 3.4.0)^49^. Matching of tumor and whole blood control samples was assessed using VCF2LR (GeneTalk) for same SNP fingerprint. Cross-sample contamination was estimated using ContEst (Broad Institute) by evaluating the likelihood of detecting alternate alleles of SNPs reported in the 1000 g database. Potential DNA damaging and sequencing artifacts from FFPE samples were filtered through rigorous quality control procedures as previously described^48^. DNA damaging was assessed using the Picard tool (http://broadinstitute.github.io/picard/) and qualified samples needed to have minimum Total QScores of 35 or contamination rates less than 0.02. Somatic SNV and insertion/deletions (INDELs) were called using Vardict (V 1.5.4). SNVs and INDELs were further filtered using previously reported criteria: (i) <4 supporting reads or <2% variant allele frequency (VAF); (ii) present in >1% population frequency in the 1000G^50^ or ExAC^51^ database; and (iii) appear on an internally collected list of recurrent sequencing errors (≥3 variant reads and ≤20% VAF in at least 30 of 2000 normal samples) on the Illumina HiSeq 4000 platform. Final mutations were annotated using vcf2maf.

### Mutational signature analysis

Both silent and non-silent mutations were extracted for mutational signature analysis using the R package sigminer (v1.2.1)^52^. Single base substitutions (SBS) and small indels and deletions (ID) in the COSMIC database (v3.1) were used as reference mutational signatures. The SBS signatures were categorized into 12 different groups according to their aetiologies^53^. Mutations that possibly contribute to sequencing artifacts were not considered for analysis (i.e., SBS27, SBS43, SBS45 to SBS60).

### Copy number analysis

Copy number analysis was performed using FACETS (Ver 0.5.13). Somatic CNA events were assigned based on sample-ploidy values calculated in the FACETS algorithm^54^. For arm-level CNV events, 39 autosomal chromosome arms were assessed. Specifically, arm-level CNA gain (>sample average ploidy +1) was defined if segments of amplification and deep amplification events account for more than 60% of total segments for the corresponding chromosome arm. Similarly, arm-level CNA loss (<sample average ploidy -1) was identified if segments of deletion and deep deletion events account for more than 60% of total segments for the given chromosome. For gene-level CNV events, only deep amplifications and deep deletions segments were considered. The fraction of genome altered (FGA) was calculated as the average proportion of segments with copy number alterations defined above across all autosomal chromosome arms.

### Reconstruction of clonal/subclonal architecture and tumor evolution

Tumor purity was estimated using ABSOLUTE^55^. Cancer cell fraction (ccf) of mutations were estimated with Pyclone^56^ and FACETS^57^. Pyclone was used to estimate major clones and subclones and reconstruct the phylogenetic tree. Cellular prevalence (CP) was estimated based on allele frequency and copy numbers to adjust for tumor purities and was used for mutation clustering. For each dissected or single tumor regions, mutations with ccf >0.6 were considered as clonal events and otherwise subclonal. SCHISM^58^ was then used to reconstruct clonal and subclonal hierarchy. The intratumoral heterogeneity (ITH) of a tumor sample was computed as previously described^59^, where the cluster with largest cancer cell fraction (ccf) was considered as the major clone, ${C}_{{main}}$. In cases where only one mutation was detected in this cluster, it was combined with the cluster containing mutations with the second largest ccf. All other clusters were pooled as the subclones ${C}_{{sub}}$. ITH was then calculated as the proportion of subclonal mutations using the following formula:

$${ITH}=\frac{{\rm{N}}({C}_{{sub}})}{{\rm{N}}\left({C}_{{main}}\right)+{\rm{N}}({C}_{{sub}})}$$

Where ${\rm{N}}$ represents the number of mutations detected in this major or subclonal clusters. The Phangorn package was then utilized to reconstruct phylogenetic trees of tumor samples based on the maximum parsimony approach^60^.

### Tissue processing for Stereo-sequencing

Two consecutive tissue sections of 10 μm were trimmed. One slide was stained by H&E staining following previous protocol^61^. The second slide was adhered to the Stereo-seq chip surface and incubated at 37 °C for 3–5 min. Then, the sections were fixed in methanol and incubated for 40 min at −20 °C. Stereo-seq library preparation and sequencing followed previously published protocol^17^.

### In situ reverse transcription

Prepared section was processed according to the Stereo-seq Transcriptomics Set User Manual (STOmics) and all reagents were from the Stereo-seq Transcriptomics T kit and Stereo-seq Library Preparation kit (STOmics)^17^. Briefly, after being washed with 0.1×SSC buffer supplemented with 0.05 U/μl RNase inhibitor, tissue sections placed on the chip were permeabilized with 1× permeabilization reagent (diluted by 0.01 N HCL) at 37 °C for 10 min (Permeabilization time was determined by the Stereo-seq Permeabilization Kit, STOmics). RNA released from the permeabilized tissue and captured by the DNB was reversely transcribed overnight at 42 °C with RT Mix (Stereo-seq Transcriptomics T kit, STOmics). After reverse transcription, tissue sections were washed with PR rinse buffer (Stereo-seq Transcriptomics T kit, STOmics) and digested with Tissue Removal buffer (Stereo-seq Transcriptomics T kit, STOmics) at 37 °C for 30 min. cDNA-containing chips were then washed with PR rinse buffer. The cDNA was then amplified as follows: incubation at 95 °C for 5 min, 15 cycles at 98 °C for 20 s, 58 °C for 20 s, 72 °C for 3 min and a final incubation at 72 °C for 5 min.

### Stereo-seq library preparation and sequencing

The concentrations of the resulting PCR products were quantified by Qubit™ dsDNA HS Assay Kit (ThermoFisher, Q32854). A total of 20 ng of DNA were then fragmented with TME (Stereo-seq Library Preparation kit, STOmics) at 55 °C for 10 min, after which the reactions were then ceased with Stop Buffer (Stereo-seq Library Preparation kit, STOmics). Fragmented products were amplified with PCR Library Mix (Stereo-seq Library Preparation kit, STOmics) as follows1 cycle of incubation at 95 °C for 5 min, 13 cycles at 98 °C for 20 s, 58 °C for 20 s and 72 °C for 30 s, and a final incubation at 72 °C for 5 min. The PCR products were purified using the AMPure XP Beads (0.6× and 0.15×) (Stereo-seq Library Preparation kit, STOmics). The concentration of purified PCR product was measured with Qubit dsDNA HS Kit (ThermoFisher, Q32854). The DNB was prepared by High-throughput Sequencing Primer Kit (STOmics) and sequenced on MGI DNBSEQ-Tx sequencer.

### Spatial profiling and trajectory analysis

Fastq files were generated using a MGI SEQ-2000 sequencer. Then if the reverse reads consist of the cDNA sequences, the synthesized coordinate identifiers (CID) and molecular identifiers (MID) were included in the forward reads (CID: 1–25 bp, MID: 26-35 bp). CID sequences on the forward reads were first mapped to the designed coordinates of the in situ captured chip with 1 base mismatch to correct for sequencing and PCR errors. Then filtered out the reads with MID containing either N bases or more than two bases with quality score lower than 10. CID and MID associated with each read were appended to each read header. Retained reads were then aligned to the reference genome (GRCh38) using STAR^62^ and mapped reads with MAPQ were counted and annotated to their corresponding genes using an in-house script (available at https://github.com/BGIResearch/handleBam). MID with the same CID and the same gene locus collapsed. Finally, this information was used to generate a CID-containing expression profile matrix.

The expression profile matrix was divided into non-overlapping bins covering an area of 100 × 100 DNB with stereopy (available at https://github.com/BGIResearch/stereopy). Then the binned spots with less than 30 genes and genes expressed in less than 30 spots were filtered. Subsequently, data normalization and unsupervised clustering were performed using scanpy^63^ with normalize_total, log1p and scale functions. The principal component analysis (PCA) was applied for the dimension reduction. With these settings, we ran the Uniform Manifold Approximation and Projection (UMAP) algorithm to obtain two-dimensional data projections, followed by Leiden clustering to identify all clusters within the dataset. We determined the marker genes for the clusters of interest using the FindMarkers function in Seurat^64^ with the following parameters: min.pct = 0.25, logfc.threshold = 0.25. Finally, we chose the genes with *P* value < 0.01 and logfc.threshold>2 as the marker genes of the trajectory analysis.

To define the developmental trajectory of cells, we performed pseudo-temporal ordering of individual cells by Monocle2^65^. Subsample was performed to randomly select 30,000 cells from the total cells. Then, after identifying the variable features in the normalized expression profile matrixes of the integrated data, the DDRTree method was used to construct the pseudotime developmental trajectories.

### Mouse model

The xenograft model was developed as previously described^66^. Briefly, malignant tumor cells of an ASC patient with *MET e*xon 14 skipping were isolated from biopsied tumor tissue. Female NOD SCID mice (Beijing Vital River Laboratory Animal Technology Co., Ltd) of 6–8 weeks of age were used for implantation. Mice were hosted in a specific pathogen-free (SPF) environment of a vivarium facility for at least three days before initiation of any experiments following IACUC protocols. Patient tumor specimens were then implanted subcutaneously in the right flanks of six mice using an 18-gauge trocar needle. The inoculated tumor grew in the host for 2–4 months before the graft was taken for analysis and grafting in the next passage. The first implanted passage was defined as P0 mice. The P0 tumor was subsequently implanted in the next passage defined as P1 mice. Total of four passages were generated by serial implantation to observe the transformation of tumor morphology (P1-P4). For each generation, tumors resected from the mice were evaluated by H&E staining and IHC for the expression of TTF1 and P40 markers. The xenograft experiment has been reviewed and approved by the Shanghai Chest Hospital Research Ethics Committee (KS1950) and the patient has signed written informed consent for the experiment.

### Statistical analysis

Between-group difference of genomic alterations was compared by Fisher’s exact test. The association of different mutation signatures in different lung cancer molecular subtypes was compared with the Wilcoxon rank-sum test. Clinical efficacy of EGFR-TKI treatment was assessed with progression-free survival, defined as the length from treatment initiation to time of disease progression or death. Kaplan–Meier estimator was used to compute patient survival overtime and the log-rank test was used to compare survival differences. Two-sided *P* values < 0.05 were considered statistically significant.

### Reporting summary

Further information on research design is available in the Nature Research Reporting Summary linked to this article.

### Supplementary information

Supplemental Materials

Reporting Summary

## Data Availability

Public WES data of Asian lung adenocarcinoma and lung squamous cell carcinoma can be acquired from the cBioPortal website (https://www.cbioportal.org/study?id=luad_oncosg_2020) and the TCGA Research Network (https://portal.gdc.cancer.gov/). Whole-exome sequencing data of ASC patients and Stereo-seq data can be accessed from GSA-human (https://ngdc.cncb.ac.cn/gsa-human/) under Project HRA004240. Whole-exome sequencing data of previous PDX study was obtained from BioProject with accession code PRJNA765468.

## Code availability

No custom code was used in this study.
