# Supplementary material for: Clonal dynamics and Stereo-seq resolve origin and phenotypic plasticity of adenosquamous carcinoma
Source: NPJ Precis Oncol. 2023 Aug 26;7:80. doi: 10.1038/s41698-023-00430-8 (PMC10460394; doi:10.1038/s41698-023-00430-8)
Supplement: Supplementary file 1 — Supplemental Materials [file 41698_2023_430_MOESM1_ESM.pdf]

Clonal dynamics and stereo-seq resolve origin and phenotypic plasticity of adenosquamous carcinoma

SUPPLEMENTARY FIGURES

Supplementary Figure 1. Schematic illustration of the patient cohorts.
ASC, Adenosquamous carcinoma; LUAD, lung adenocarcinoma; LUSC, lung squamous cell carcinoma; *EGFR*, epidermal growth factor receptor.

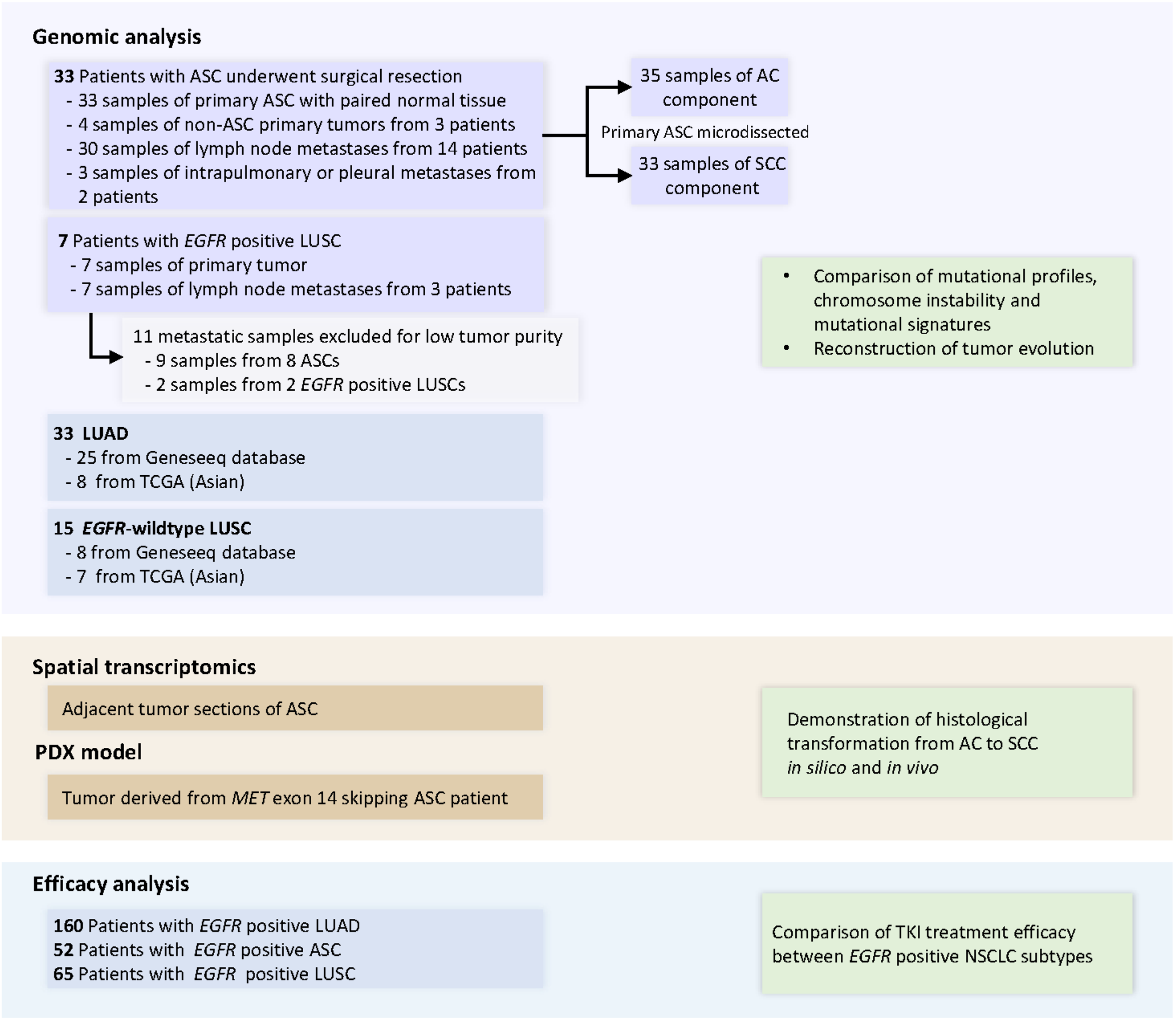

Supplementary Figure 2. Venn diagrams showing heterogeneity between AC and SCC components of microdissected ASCs.

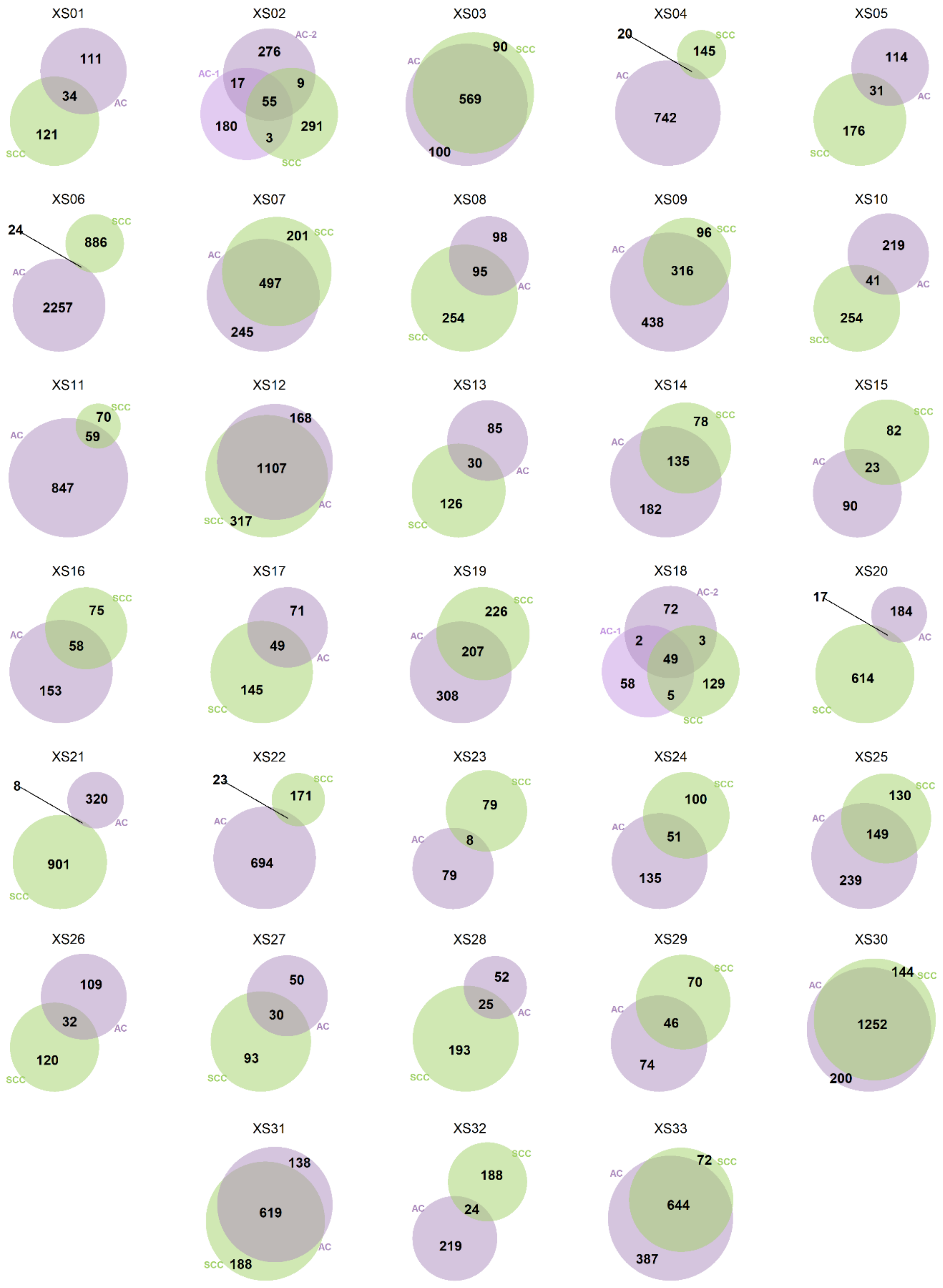

Supplementary Figure 3. Comparison of tumor mutational burden (TMB) and intratumoral heterogeneity (ITH) between primary and metastatic sites, and in NSCLC subtypes. a TMB compared between primary and LN samples. **b** ITH compared between primary and LN samples of EGFR-positive ASC. **c** ITH compared among NSCLC subtypes. **d** ITH compared among EGFR-positive LUADs, ASCs, and LUSCs. The centre lines of the boxes represent the median value of each covariate. The lower and upper edges of the boxes correspond to the first and third quartiles and whiskers defines the range within 1.5 times the interquartile range (IQR) from the edges of the boxplot. Dots indicate outliers, defined as data points that fall outside 1.5 times the IQR. Two-sided P values were derived using Wilcoxon rank-sum test.

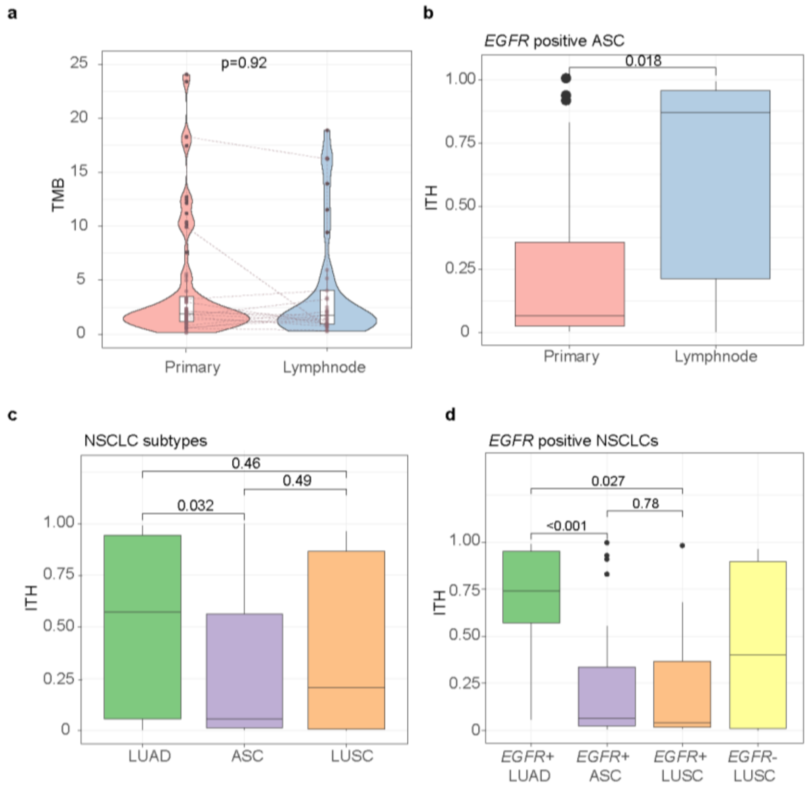

Supplementary Figure 4. Mutational signatures associated with different molecular subtypes of ASC. Mutational signatures of **a** *EGFR* or **b** MET- driven lung cancer subtypes were estimated using single base substitutions in these tumors. The centre lines of the boxes represent the median value of each covariate. The lower and upper edges of the boxes correspond to the first and third quartiles and whiskers defines the range within 1.5 times the interquartile range (IQR) from the edges of the boxplot. Dots indicate outliers, defined as data points that fall outside 1.5 times the IQR. Two-sided P values were derived using Wilcoxon rank-sum test.

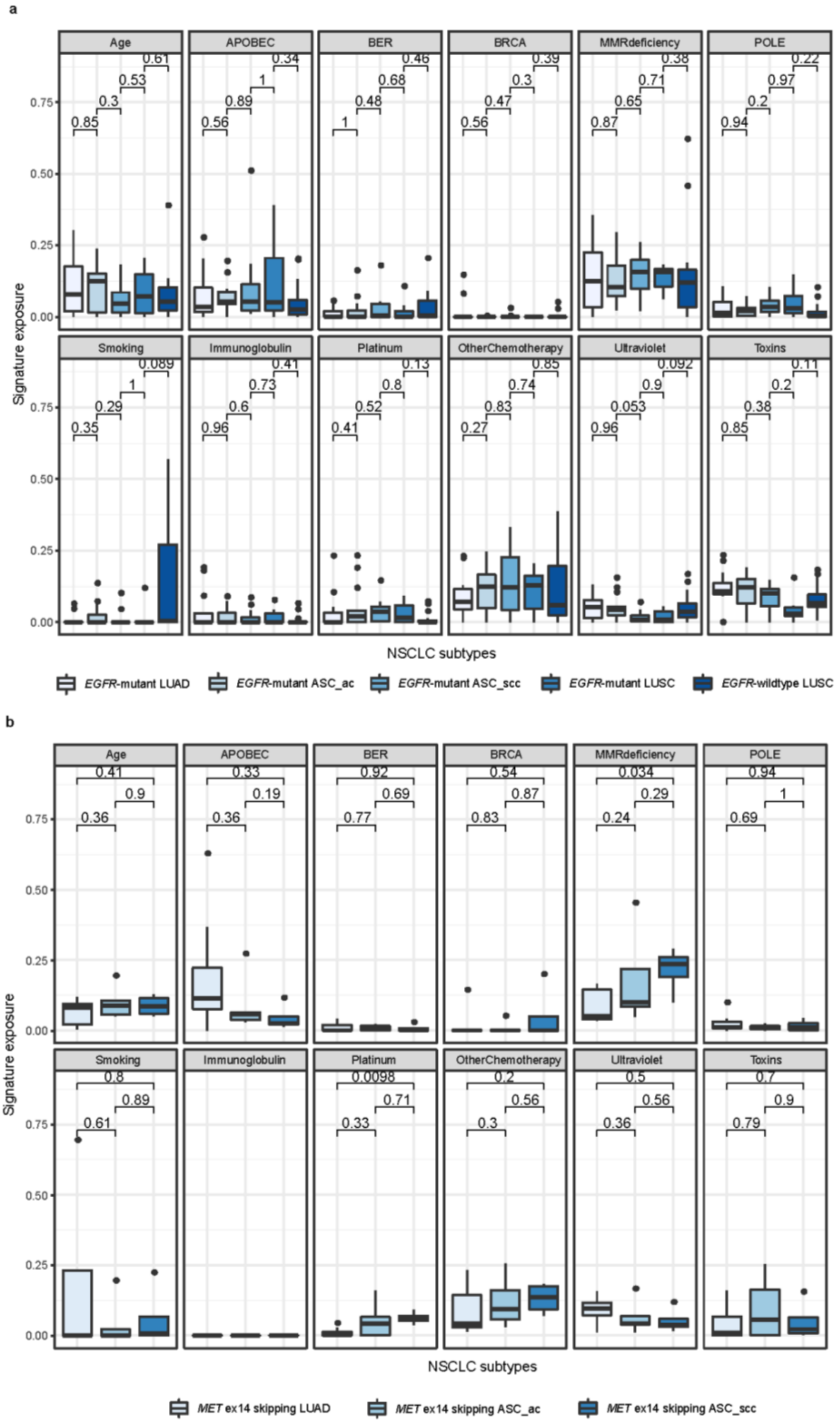

Supplementary Figure 5. Evolutionary tracks of EGFR positive ASCs. Schematic diagram showing the positions of lesions taken from ASC Patients XS29 (*EGFR* 19del), XS18 (*EGFR* L861Q), XS22 (*EGFR* 19del), XS27 (*EGFR* 19del), and XS23 with labeled histological subtypes and corresponding clonal phylogenies of key driver mutations. Histological subtypes: ADC, adenocarcinoma; ASC, adenosquamous carcinoma; GGO, ground-glass opacity; LN, lymph node metastases.

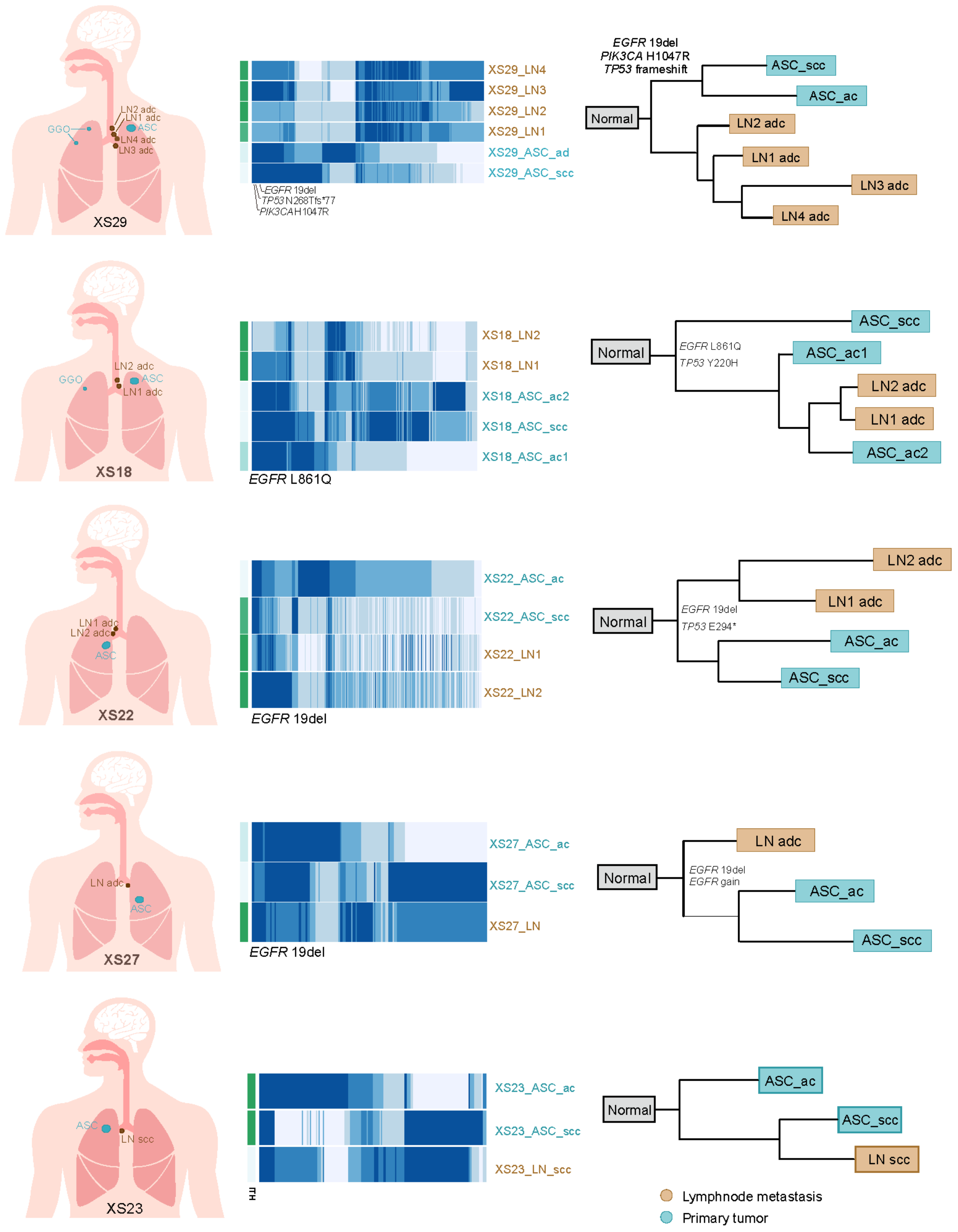

Supplementary Figure 6. Reconstruction of evolutionary phylogeny in PDX model. a Venn diagram showing number of shared and unique somatic mutations in untreated patient primary ASC and untreated mice subcutaneous tumor from BioProject PRJNA765468. This study established PDX model from an ASC patient with *MET* exon 14 skipping mutation. **b** Reconstructed phylogenetic tree showing clonal relationships and cancer cell fractions selected trunk and branch mutations.

a

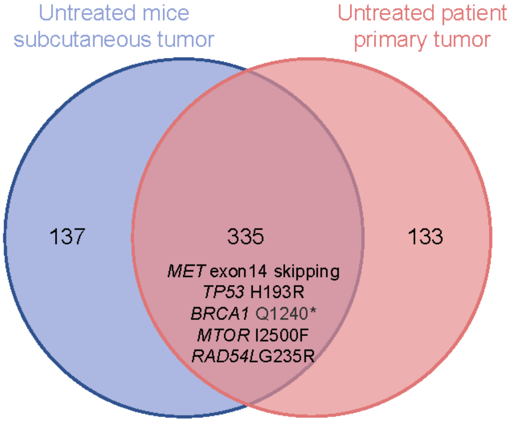

b

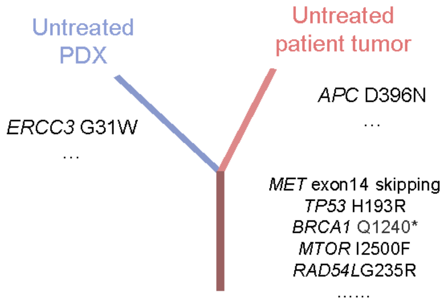

Mutant	Untreated PDX	Untreated patient tumor
MET ex14 skipping	0.97	0.73
TP53 H193R	0.99	0.73
MTOR I2550F	0.41	0.27
BRCA1 Q1240*	0.45	0.33
RAD54L G235R	0.4	0.29
ERCC3 G31W	0.15	<0.05
APC D396N	<0.05	0.19

Supplementary Table 1. Levels of copy number variation in NKX2-1 containing chromosome segments

PID	Microdissected_sample	Gene	Chr	Seg_start	Seg_end	CNV_stat	Level
XS01	ASC_ac	*NKX2-1*	14	24877300	42355900	Amplification	1
XS01	ASC_scc	*NKX2-1*	14	33014500	68249300	Amplification	1
XS02	ASC_ac_H	*NKX2-1*	14	33014500	105953000	Amplification	1
XS02	ASC_ac_L	*NKX2-1*	14	31081500	68280800	Amplification	1
XS02	ASC_scc	*NKX2-1*	14	30135500	77580700	Neutral	0
XS03	ASC_ac	*NKX2-1*	14	24458211	53521300	Neutral	0
XS03	ASC_scc	*NKX2-1*	14	24544400	105943679	Deletion	-1
XS04	ASC_ac	*NKX2-1*	14	29237600	105406500	Amplification	1
XS04	ASC_scc	*NKX2-1*	14	30194900	105964300	Neutral	0
XS05	ASC_ac	*NKX2-1*	14	19110200	105996119	Neutral	0
XS05	ASC_scc	*NKX2-1*	14	19110100	105996119	Neutral	0
XS06	ASC_ac	*NKX2-1*	14	23939000	68276000	Neutral	0
XS06	ASC_scc	*NKX2-1*	14	24807800	105965000	Deletion	-1
XS07	ASC_ac	*NKX2-1*	14	24877300	91143000	Neutral	0
XS07	ASC_scc	*NKX2-1*	14	19110200	100848100	Deletion	-1
XS08	ASC_ac	*NKX2-1*	14	33293600	57945100	Neutral	0
XS08	ASC_scc	*NKX2-1*	14	24563900	59970500	Neutral	0
XS09	ASC_ac	*NKX2-1*	14	31062800	105942800	Neutral	0
XS09	ASC_scc	*NKX2-1*	14	31066700	105963700	Neutral	0
XS10	ASC_ac	*NKX2-1*	14	24552000	93686600	Neutral	0
XS10	ASC_scc	*NKX2-1*	14	20444588	78189600	Neutral	0
XS11	ASC_ac	*NKX2-1*	14	19110200	105962234	Deletion	-1
XS11	ASC_scc	*NKX2-1*	14	24723500	78227600	Deletion	-1
XS12	ASC_ac	*NKX2-1*	14	20404238	39901300	Amplification	1
XS12	ASC_scc	*NKX2-1*	14	19110200	105996119	Neutral	0
XS13	ASC_ac	*NKX2-1*	14	19118200	75131600	Deletion	-1
XS13	ASC_scc	*NKX2-1*	14	19118200	105995534	Neutral	0
XS14	ASC_ac	*NKX2-1*	14	19110000	105996119	Amplification	1
XS14	ASC_scc	*NKX2-1*	14	19110000	105996119	Neutral	0
XS15	ASC_ac	*NKX2-1*	14	24523000	45679500	Amplification	1
XS15	ASC_scc	*NKX2-1*	14	19110200	105996119	Deletion	-1
XS16	ASC_ac	*NKX2-1*	14	36943100	38311400	Amplification	1
XS16	ASC_scc	*NKX2-1*	14	21502300	51111300	Neutral	0
XS17	ASC_ac	*NKX2-1*	14	24838900	75151400	Deletion	-1
XS17	ASC_scc	*NKX2-1*	14	24974800	75130700	Deletion	-1
XS18	ASC_ac1	*NKX2-1*	14	19110200	105962369	Neutral	0
XS18	ASC_ac2	*NKX2-1*	14	30093400	105639300	Neutral	0
XS18	ASC_scc	*NKX2-1*	14	30093300	105939672	Deletion	-1
XS19	ASC_ac	*NKX2-1*	14	24976700	45334700	Amplification	1
XS19	ASC_scc	*NKX2-1*	14	25102300	45334700	Neutral	0

PID	Microdissected_sample	Gene	Chr	Seg_start	Seg_end	CNV_stat	Level
XS20	ASC_ac	*NKX2-1*	14	26917500	92505900	Neutral	0
XS20	ASC_scc	*NKX2-1*	14	19118200	105995600	Neutral	0
XS21	ASC_ac	*NKX2-1*	14	24542700	50736000	Amplification	1
XS21	ASC_scc	*NKX2-1*	14	24838800	93299600	Amplification	1
XS22	ASC_ac	*NKX2-1*	14	19377700	105958500	Neutral	0
XS22	ASC_scc	*NKX2-1*	14	19377700	105958600	Neutral	0
XS23	ASC_ac	*NKX2-1*	14	24974800	70040100	Neutral	0
XS23	ASC_scc	*NKX2-1*	14	19110100	70713300	Neutral	0
XS24	ASC_ac	*NKX2-1*	14	24974800	75283700	Amplification	1
XS24	ASC_scc	*NKX2-1*	14	19110200	94775900	Deletion	-1
XS25	ASC_ac	*NKX2-1*	14	34904600	39900800	Amplification	1
XS25	ASC_scc	*NKX2-1*	14	31562100	105933000	Neutral	0
XS26	ASC_ac	*NKX2-1*	14	20084400	81651900	Neutral	0
XS26	ASC_scc	*NKX2-1*	14	19110000	105996119	Neutral	0
XS27	ASC_ac	*NKX2-1*	14	24976600	105996100	Neutral	0
XS27	ASC_scc	*NKX2-1*	14	25044400	105996119	Amplification	1
XS28	ASC_ac	*NKX2-1*	14	24974800	75151500	Amplification	1
XS28	ASC_scc	*NKX2-1*	14	19110100	105962369	Neutral	0
XS29	ASC_ac	*NKX2-1*	14	19110000	105962369	Neutral	0
XS29	ASC_scc	*NKX2-1*	14	19110100	105962369	Deletion	-1
XS30	ASC_ac	*NKX2-1*	14	19110100	105996119	Neutral	0
XS30	ASC_scc	*NKX2-1*	14	19110100	105996119	Amplification	1
XS31	ASC_ac	*NKX2-1*	14	19110100	105962369	Deletion	-1
XS31	ASC_scc	*NKX2-1*	14	19110100	105962369	Deletion	-1
XS32	ASC_ac	*NKX2-1*	14	20848400	105407900	Amplification	1
XS32	ASC_scc	*NKX2-1*	14	19377907	105995400	Amplification	1
XS33	ASC_ac	*NKX2-1*	14	19110200	105962369	Deletion	-1
XS33	ASC_scc	*NKX2-1*	14	19110200	105962300	Deletion	-1

Supplementary Table 2. Clinical characteristics of the TKI-treated LUAD, ASC, and LUSC patients

	**Adenocarcinoma**	**Adenosquamous carcinoma**	**Squamous cell carcinoma**
n	160	52	65
**Age**			
median	61	61	63
range	37-84	39-80	37-83
**Sex**			
Female	97 (61%)	30 (58%)	34 (52%)
Male	63 (39%)	22 (42%)	31 (48%)
**EGFR subtype**			
exon 19 deletion	78 (49%)	23 (44%)	36 (55%)
L858R	82 (51%)	24 (46%)	29 (45%)
T790M	0	5 (10%)	0
**TKI used as 1L treatment**	**131 (82%)**	**25 (48%)**	**44 (68%)**
1st generation TKI	118	23	38
2nd generation TKI	1	0	2
3rd generation TKI	4	0	0
TKI combination regimens	8	2	4
**TKI used as 2L treatment**	**28 (18%)**	**18 (35%)**	**19 (29%)**
1st generation TKI	26	15	14
2nd generation TKI	0	2	4
3rd generation TKI	1	0	1
TKI combination regimens	1	1	0
**TKI used as 3L treatment**	**1 (1%)**	**9 (17%)**	**2 (3%)**
1st generation TKI	1	6	1
2nd generation TKI	0	0	1
3rd generation TKI	0	1	0
TKI combination regimens	0	2	0
