# Supplementary material for: Clonal dynamics and Stereo-seq resolve origin and phenotypic plasticity of adenosquamous carcinoma
Source: NPJ Precis Oncol. 2023 Aug 26;7:80. doi: 10.1038/s41698-023-00430-8 (PMC10460394; doi:10.1038/s41698-023-00430-8)
Supplement: Supplementary file 2 — Reporting Summary [file 41698_2023_430_MOESM2_ESM.pdf]

nature portfolio

Corresponding author(s): Yuchen Han and Shun Lu

Last updated by author(s): May 5, 2023

Reporting Summary

Nature Portfolio wishes to improve the reproducibility of the work that we publish. This form provides structure for consistency and transparency in reporting. For further information on Nature Portfolio policies, see our Editorial Policies and the Editorial Policy Checklist.

Statistics

For all statistical analyses, confirm that the following items are present in the figure legend, table legend, main text, or Methods section.

n/a	Confirmed	
☐	☒	The exact sample size (*n*) for each experimental group/condition, given as a discrete number and unit of measurement
☒	☐	A statement on whether measurements were taken from distinct samples or whether the same sample was measured repeatedly
☐	☒	The statistical test(s) used AND whether they are one- or two-sided<br>*Only common tests should be described solely by name; describe more complex techniques in the Methods section.*
☐	☒	A description of all covariates tested
☒	☐	A description of any assumptions or corrections, such as tests of normality and adjustment for multiple comparisons
☐	☒	A full description of the statistical parameters including central tendency (e.g. means) or other basic estimates (e.g. regression coefficient) AND variation (e.g. standard deviation) or associated estimates of uncertainty (e.g. confidence intervals)
☐	☒	For null hypothesis testing, the test statistic (e.g. *F*, *t*, *r*) with confidence intervals, effect sizes, degrees of freedom and *P* value noted<br>*Give P values as exact values whenever suitable.*
☒	☐	For Bayesian analysis, information on the choice of priors and Markov chain Monte Carlo settings
☒	☐	For hierarchical and complex designs, identification of the appropriate level for tests and full reporting of outcomes
☒	☐	Estimates of effect sizes (e.g. Cohen's *d*, Pearson's *r*), indicating how they were calculated

Our web collection on statistics for biologists contains articles on many of the points above.

Software and code

Policy information about availability of computer code

Data collection	No software was used for data collection.
Data analysis	WES sequencing data were analyzed using Trimmomatic, Burrows-Wheeler Aligner, Picard, Genome Analysis Toolkit (GATK 3.4.0), Vardict (V1.5.4), ABSOLUTE, Pyclone, and FACETS (Ver 0.5.13). Stereo-sequencing data were analyzed using Spliced Transcripts Alignment to a Reference (STAR v2.7.10b), and the in-house handleBam (https://github.com/BGIResearch/handleBam). R packages Seurat and Monocle 2 were used for single-cell RNA expression and pseudotime trajectory analysis, respectively. All statistical analyses were performed in R version 4.0.3.

For manuscripts utilizing custom algorithms or software that are central to the research but not yet described in published literature, software must be made available to editors and reviewers. We strongly encourage code deposition in a community repository (e.g. GitHub). See the Nature Portfolio guidelines for submitting code & software for further information.

Data

Policy information about availability of data

All manuscripts must include a data availability statement. This statement should provide the following information, where applicable:

- Accession codes, unique identifiers, or web links for publicly available datasets
- A description of any restrictions on data availability
- For clinical datasets or third party data, please ensure that the statement adheres to our policy

Public WES data of Asian lung adenocarcinoma and lung squamous cell carcinoma can be acquired from the cbioportal website (https://www.cbioportal.org/study?id=luad_oncosg_2020) and the TCGA Research Network (https://portal.gdc.cancer.gov/). Whole-exome sequencing data of ASC patients and stereo-seq data can be accessed from GSA-human (https://ngdc.cncb.ac.cn/gsa-human/) under Project HRA004240. Whole-exome sequencing data of previous PDX study was obtained from BioProject with accession code PRJNA765468.

Research involving human participants, their data, or biological material

Policy information about studies with human participants or human data. See also policy information about sex, gender (identity/presentation), and sexual orientation and race, ethnicity and racism.

Reporting on sex and gender	Sex and gender were not specifically examined in this study.
Reporting on race, ethnicity, or other socially relevant groupings	All patient included in this study were of Chinese ethnicity. For TCGA data, only East Asian patients were selected for analysis.
Population characteristics	This study included 33 patients with adenosquamous carcinoma, ranging from 44 to 78 years of age. Among these, two were known smokers, two were former smokers, 27 were non-smokers, and two were unknown of smoking history. Twenty patients carry driver mutations, including EGFR (exon 19 deletion, L858R, or L861Q), MET exon 14 skipping, KRAS G12C/D, and ERBB2 exon 20 insertion. Other patients did not carry clearly defined lung cancer drivers. Additionally, seven patients with lung squamous cell carcinoma were included. All were positive for EGFR sensitive mutations.
Recruitment	Archived FFPE samples of patients with adenosquamous carcinoma were retrospectively reviewed and those passed pathological confirmation were included for this study.
Ethics oversight	This study was reviewed and approved by the Shanghai Chest Hospital Research Ethics Committee.

Note that full information on the approval of the study protocol must also be provided in the manuscript.

Field-specific reporting

Please select the one below that is the best fit for your research. If you are not sure, read the appropriate sections before making your selection.

☒ Life sciences ☐ Behavioural & social sciences ☐ Ecological, evolutionary & environmental sciences

For a reference copy of the document with all sections, see nature.com/documents/nr-reporting-summary-flat.pdf

Life sciences study design

All studies must disclose on these points even when the disclosure is negative.

Sample size	Archived FFPE samples of patients diagnosed with adenosquamous carcinoma at our center were retrospectively reviewed and all those passed pathological confirmation were included for this study.
Data exclusions	No data were excluded from analyses.
Replication	Replication was not applicable for this retrospective clinical study.
Randomization	No randomization was done as this was a retrospective study.
Blinding	Blinding was not applicable for this retrospective clinical study.

Reporting for specific materials, systems and methods

We require information from authors about some types of materials, experimental systems and methods used in many studies. Here, indicate whether each material, system or method listed is relevant to your study. If you are not sure if a list item applies to your research, read the appropriate section before selecting a response.

Materials & experimental systems

n/a	Involved in the study	
☐	☒	Antibodies
☒	☐	Eukaryotic cell lines
☒	☐	Palaeontology and archaeology
☐	☒	Animals and other organisms
☒	☐	Clinical data
☒	☐	Dual use research of concern
☒	☐	Plants

Methods

n/a	Involved in the study	
☒	☐	ChIP-seq
☒	☐	Flow cytometry
☒	☐	MRI-based neuroimaging

Antibodies

Antibodies used	TTF-1 (Cell Signaling Technology); p40 (ZSGB-BIO)
Validation	TTF-1 and p40 expression were evaluated by two experienced clinical pathologists. Positive and negative controls were referred to read the results.

Animals and other research organisms

Policy information about studies involving animals; ARRIVE guidelines recommended for reporting animal research, and Sex and Gender in Research

Laboratory animals	For patient-derived xenograft model, female NOD SCID mice (Beijing Vital River Laboratory Animal Technology Co., Ltd) of 6-8 weeks of age were used for implantation.
Wild animals	The study did not involve wild animals.
Reporting on sex	Sex was not considered as a factor of this study.
Field-collected samples	The study did not involve field-collected samples.
Ethics oversight	All animal experiments were conducted following the The Animal Study Protocol (IACUC).

Note that full information on the approval of the study protocol must also be provided in the manuscript.
